# Towards secure and efficient integration of blockchain and 6G networks

**DOI:** 10.1371/journal.pone.0302052

**Published:** 2024-04-11

**Authors:** Yang Liu, Song Peng, Miaomiao Zhang, Shidong Shi, Jianhao Fu

**Affiliations:** School of Information Science and Engineering, Henan University of Technology, Zhengzhou, Henan, China; Universiti Teknologi Malaysia, MALAYSIA

## Abstract

The future of communication systems is undergoing a transformative shift towards intelligence, efficiency, and flexibility. Presently, the amalgamation of blockchain technology and the sixth-generation mobile communication network (6G) has garnered significant attention, as their fusion is poised to profoundly impact the digital economy and society at large. However, the convergence of blockchain and 6G networks poses challenges pertaining to security and performance. In this article, we propose an approach based on the design of secure mechanisms and performance optimization to delve into the key issues surrounding the integration of blockchain and 6G networks from both security and performance perspectives. Specifically, we first introduce the application scenarios of 6G networks and blockchain’s empowerment of them to highlight the necessity of combining blockchain technology with 6G. Subsequently, in order to ensure the security of communication and data transmission between blockchain and 6G networks, we have investigated the design requirements for security mechanisms. Furthermore, we discuss the efficient realization of the amalgamation between blockchain and 6G networks by proposing a solution based on Directed Acyclic Graph (DAG) for blockchain’s asynchronous consensus protocol, alongside optimization strategies for storage and communication to meet the desired characteristics and requirements of 6G networks. Lastly, we provide valuable research directions that serve as references and guidance for the future development of the integration between blockchain and 6G networks.

## Introduction

The 6G network is the next generation of wireless communication technology, built upon the foundation of the 5G network [1–3]. Compared to 5G, 6G offers higher spectrum efficiency, lower latency, faster data rates, broader coverage, and the ability to connect a larger number of devices [4–6]. The development of 6G networks aims to establish a seamless global connectivity infrastructure, enabling efficient interconnection of all devices and systems [7]. Its technical architecture will rely on smarter technologies such as Software-Defined Networking (SDN), Network Function Virtualization (NFV), Mobile Edge Computing (MEC), big data, artificial intelligence (AI), and quantum communication to deliver more efficient, secure, and reliable communication services [8].

The 6G network is dedicated to a user-centric approach, emphasizing user experience, and excelling in network performance, service quality, and application scenarios [9]. Furthermore, the 6G network possesses robust heterogeneous support capabilities, enabling multimodal connections that cover different spectrums, network types, and devices [10]. From a technological perspective, the 6G network will be a highly heterogeneous network comprising numerous small intelligent devices and sensors. It will find applications in various scenarios such as the Internet of Things (IoT), smart cities, intelligent manufacturing, healthcare, and autonomous driving. The overarching goal is to achieve ubiquitous connectivity, comprehensive coverage, extensive perception, and complete intelligence. Additionally, the development of the 6G network will drive technological innovation and industrial upgrading in several domains, including novel antenna technologies, terahertz communications, and artificial intelligence [11].

However, in the evolving trends of the digital information era, 6G networks are moving towards a higher degree of decentralization. Yet, in this process, they face a series of intricate issues such as trust establishment, resource sharing, secure data exchange, access control, privacy protection, and effective governance [8]. Traditional network security techniques often rely on centralized institutions and encryption algorithms to address data security, transparency, and trust issues. However, in the large-scale distributed systems of the 6G environment, these methods may not meet the requirements of real-time operation, scalability, and decentralization. At this juncture, the unique advantages of blockchain technology come to the fore: its decentralization, immutability, and traceability offer potential solutions to address these challenges, promising to significantly enhance the security and reliability of 6G networks [12].

The integration of blockchain with 6G networks not only effectively addresses the information security demands of the new era but also has the potential to catalyze entirely new digital economic and social models, leading to profound societal impacts [13–15]. However, this convergence also faces a series of technical challenges and issues, such as security and privacy protection, performance and scalability of consensus mechanisms, and efficient and low-latency data and communication [16]. The motivation of this paper is precisely based on this backdrop, aiming to design a solution that combines security mechanisms with performance optimization. This solution seeks to ensure the secure and efficient integration of blockchain technology within the 6G network environment. The main contributions of this article are as follows:

- We are committed to exploring how to securely and efficiently integrate blockchain technology in 6G networks. By proposing methods based on secure mechanism design and performance optimization, we have conducted in-depth analyses of the application of blockchain technology in 6G networks, focusing on both security and performance optimization aspects, and put forward feasible solutions.
- We have conducted an in-depth study of the design requirements for security mechanisms. By thoroughly analyzing the security threats and attack types faced by the integration of blockchain and 6G networks, we have researched a range of design requirements for security mechanisms to ensure the security and reliability of communication and data transmission between blockchain and 6G networks.
- Our focus lies on the efficient implementation of the integration between blockchain and 6G networks. We have proposed a solution based on DAG for blockchain asynchronous consensus protocol. This solution aims to meet the desired characteristics of low latency, high throughput, and high reliability in the 6G network. Additionally, we have provided optimization strategies in various aspects such as storage and communication to accommodate the substantial data exchange and processing requirements in the 6G network.

## The applications and challenges of 6G networks

As an integral component of the next generation of communication networks, 6G possesses not only application potential in traditional communication domains but also significant roles to play in various fields such as healthcare, military, entertainment, and industry. Below, we will delve into the specific application scenarios of 6G networks in these domains and the transformative impact they bring, while also highlighting the key security and performance challenges accompanying their rapid development.

### Healthcare

6G networks have facilitated the digitization of the healthcare industry, significantly enhancing the efficiency, reliability, and security of medical services. Through remote diagnosis and surgical techniques, physicians can improve their workflow efficiency and reduce the need for in-person patient visits. Leveraging the high-speed data transmission capabilities of 6G networks, real-time analysis of patient health data allows doctors to gain a more accurate understanding of patient conditions, thereby providing more precise treatment plans. Additionally, smart wearable devices (such as smartwatches and smart clothing) utilize 6G networks for real-time monitoring of vital signs, offering unprecedented accuracy in personal health management. Nevertheless, as medical information flows rapidly across 6G networks, ensuring data security, protecting privacy, and addressing potential security threats emerge as critical challenges.

### Military

With its capabilities for massive-scale, high-speed, and real-time data processing, 6G networks have greatly enhanced the efficiency and combat capabilities of military operations. Battlefield intelligence collection and analysis can be conducted rapidly and efficiently, enabling commanders to gain a deeper understanding of the battlefield situation and make more accurate and timely decisions based on this information. However, while enhancing military effectiveness, ensuring the high security and stability of military intelligence transmission under 6G networks is crucial. Preventing potential network attacks and information leaks has become one of the core challenges in technological development.

### Entertainment culture

The entertainment and cultural industries are benefiting from 6G networks, undergoing a profound transformation from traditional models to digitization and intelligence. Particularly in the virtual reality and augmented reality industries, the high-definition and low-latency characteristics of 6G networks have elevated user experiences to new heights. However, for applications with high bandwidth demands, maintaining stable high-performance services and effectively preventing issues such as illegal access and data tampering are technical challenges that 6G networks must address.

### Industrial automation and IoT

The development of industrial automation and IoT also relies heavily on the support of 6G networks. Its high-speed and low-latency characteristics greatly drive innovation in various fields, including industrial robotics, smart manufacturing, intelligent transportation systems, smart logistics, and smart homes. However, with the massive influx of devices and data exchange, 6G networks need to continuously enhance processing capabilities and optimize resource allocation while ensuring stability and security in complex environments [17, 18]. This is a crucial challenge that needs to be overcome urgently in the practical application process.

Furthermore, with the development of 6G networks, they will provide enhanced services for fields such as remote education, smart agriculture, intelligent buildings, and tourism. For instance, 6G networks can enable real-time online interactions and virtual laboratories for remote education applications. In smart agriculture, 6G networks facilitate data collection and transmission for real-time weather monitoring, farmland data analysis, and crop growth tracking. Intelligent buildings can benefit from the connectivity of various sensors and devices through 6G networks, enabling automated control and data analysis. Moreover, 6G networks offer smoother network connections and enrich the tourism industry with enhanced online experiences, injecting new vitality into the sector. However, ensuring that 6G networks can meet the specific needs of various industries while still addressing universal security and performance optimization remains a central topic for future research and development efforts.

## Blockchain empowering 6G networks

With the rapid development of the digital economy and digital society, the application of blockchain technology in fields such as IoT, and healthcare has garnered extensive attention and exploration [19–23]. As a distributed ledger technology, blockchain achieves secure data interchange and value transfer through a decentralized approach, significantly enhancing the efficiency and transparency of information exchange. In the context of 6G networks, blockchain technology can bring higher security, transparency, and reliability to the network while enabling various new application scenarios and business models [24].

The fundamental components of blockchain encompass distributed ledger structure, cryptographic algorithms, consensus mechanisms, and smart contracts. These theoretical underpinnings collectively bolster blockchain technology to provide a higher level of security assurance for 6G networks. Specifically, by dispersing transaction records among numerous network nodes and employing encryption algorithms to ensure data integrity and immutability, blockchain significantly mitigates the risks of data fraud and tampering. Furthermore, based on consensus mechanisms, blockchain can achieve network state consistency and trustworthiness, further enhancing the network’s resilience against attacks [25].

In the context of 6G network environments, the decentralized nature of blockchain technology is paramount for enhancing network security. By incorporating network transaction records and identity verification processes into blockchain, not only does it improve transaction processing speed, but it also establishes a trust framework with highly anti-tampering characteristics. The functionality of smart contracts in blockchain further applies automatically executed security rules to the network’s operational layer, effectively preventing malicious activities and rapidly responding to various network threats [26].

Addressing the key features of 6G networks, such as ultra-low latency, extremely high-speed transmission, massive connectivity, and network intelligence, blockchain technology offers unique theoretical support and solutions. For instance, integrating blockchain’s distributed ledger enables real-time, fair, and traceable resource allocation, optimizing network resource management [27]. Through the automation of decision-making and execution mechanisms in smart contracts, network service response speed and management efficiency can be enhanced while ensuring strict control over data access and exchange authorization, thereby safeguarding user privacy and data security.

## The security mechanisms of blockchain-6G network

This section will focus on how to ensure the security requirements for the combination of blockchain and 6G networks.

### Analysis of security threats and attack types

The essence of blockchain technology lies in achieving distributed consensus through decentralization, thereby ensuring the security and reliability of transactions. However, this decentralized nature also makes blockchain networks vulnerable to malicious attacks. Take Bitcoin as an example [28], where an attacker with control over more than 51% of the network’s computational power can modify transaction records on the blockchain, effectively taking control of the entire network. Additionally, since participants in a blockchain network can be anonymous, malicious actors can exploit this anonymity to carry out attacks. For instance, they may propagate malicious or forged transactions, thereby posing threats to the reliability and security of transactions.

Furthermore, smart contracts are one of the core features of blockchain technology, enabling automated execution without the need for centralized intervention. However, smart contracts are susceptible to various vulnerabilities, such as reentrancy attacks, race conditions, integer overflow, and more. Malicious actors can exploit these vulnerabilities to carry out malicious operations, including double-spending and denial-of-service attacks, posing threats to blockchain networks. Additionally, protocol-level vulnerabilities are also a common avenue for attacking blockchain networks. Attackers can attack blockchain networks by maliciously constructing messages, tampering with messages, faking identities, etc. to gain improper benefits.

In the future 6G networks, their high speed and low latency characteristics make them potential targets for attackers. Attackers can exploit the high-speed transmission feature of 6G networks to launch various forms of network attacks. For instance, distributed denial-of-service (DDoS) attacks can leverage the high bandwidth of 6G networks to flood the network with a large volume of traffic, depleting network resources and causing network paralysis. Additionally, network phishing attacks may take advantage of the high-speed nature of 6G networks to masquerade as trustworthy entities and deceive users into submitting sensitive information. Moreover, the low latency feature of 6G networks can be exploited by attackers for real-time attacks such as intrusion attacks and port scanning.

### Security mechanism design requirements

**Fig 1:**
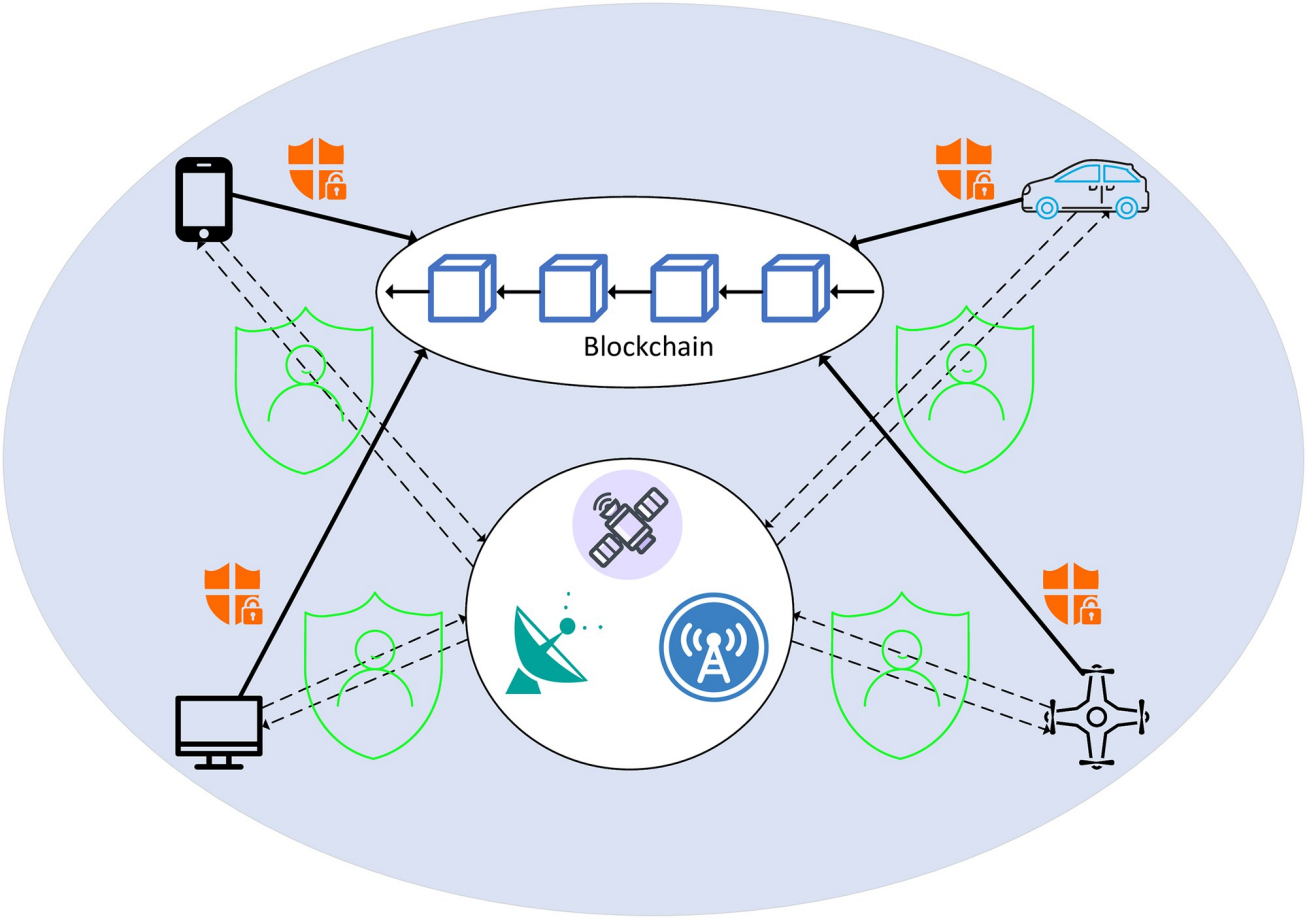
Security mechanism design requirements in blockchain-6G Network.

To ensure the security of the integration between blockchain and 6G network, it is imperative to develop a comprehensive set of security mechanisms. As depicted in Fig 1, these mechanisms encompass not only identity authentication and authorization but also encrypted communication and data privacy protection, among other aspects.

Identity authentication and authorization serve as fundamental prerequisites for the integration of blockchain and 6G networks. Within the realm of 6G networks, users can acquire network access privileges through identity authentication mechanisms, while smart contracts within the blockchain ensure compliance with established rules. In the context of applications combining blockchain and 6G networks, identity authentication mechanisms further guarantee that only authorized users can access shared data.

To ensure the data security within applications combining blockchain and 6G networks, the implementation of encryption communication mechanisms is of paramount importance. In the context of 6G networks, where wireless channels are susceptible to attacks, employing encryption communication can effectively enhance the confidentiality and integrity of transmitted data. Various encryption algorithms, such as hybrid encryption and lightweight encryption, along with key management mechanisms, can be utilized to further strengthen data security. Simultaneously, measures need to be taken to safeguard user privacy and prevent data theft or misuse. Zero-knowledge proof techniques can be employed to verify that users’ data adheres to certain rules without revealing the actual data information.

Furthermore, to prevent malicious nodes from disrupting the blockchain network, employing trust-based security mechanisms is highly necessary. Reputation assessment and trust management mechanisms are common security measures in this regard. By evaluating the reputation and trustworthiness of each node, these mechanisms can better prevent attacks from malicious nodes. Additionally, smart contracts are a vital component of blockchain networks, and thus, it is crucial to implement security measures to ensure their correctness and safety. One common approach is to utilize formal verification techniques to check for vulnerabilities and errors in smart contracts, thereby avoiding potential attacks. Furthermore, multi-signature technology can enhance the security of smart contracts and prevent the execution of malicious contracts. Lastly, conducting regular security audits and vulnerability scans is essential. This helps in identifying and promptly resolving potential vulnerabilities, thereby mitigating the risk of attackers exploiting these vulnerabilities for their malicious activities.

## Efficient implementation of blockchain-6G network

In this section, we will delve into the detailed discussion on achieving an efficient integration of blockchain and 6G networks.

### The adaptation of blockchain consensus protocols to 6G networks

In wireless networks, especially in the context of 6G communication environments, blockchain technology has demonstrated significant security advantages. However, it faces a prominent challenge of high energy consumption in its application, particularly for nodes with limited battery capacity. The complex consensus process accelerates energy consumption, which may lead to premature battery depletion and offline nodes, thereby affecting network scalability and reliability. Addressing this core issue, innovative solutions like the Green Sharding (GS) [29] scheme employ geolocation-based node sharding strategies to restrict wireless nodes’ participation in global communication, effectively reducing unnecessary energy consumption. This approach has shown promising energy efficiency and performance in scenarios involving the terahertz (THz) and the millimeter wave (mmWave) signals.

However, even with optimization solutions like GS to alleviate energy consumption issues, when integrating blockchain with 6G networks, the adaptation between blockchain consensus protocols and 6G networks is essential, as it directly affects the overall system’s performance and efficiency. Previous research endeavors aimed at integrating blockchain into wireless communication, such as employing Proof of Work (PoW) [28], Proof of Stake (PoS) [30], or Practical Byzantine Fault Tolerance (PBFT) [31] consensus protocols. However, these protocols face challenges in handling high concurrency transactions and low-latency demands [25, 29, 32]. As evident from Table 1, traditional consensus protocols typically prioritize ensuring strong consistency at the expense of lower throughput, higher latency, and poorer scalability. Particularly in wireless network environments, the synchronous or semi-synchronous communication models and high-energy consumption characteristics required by these protocols make them difficult to adapt to the large-scale connectivity, low latency, and high energy-efficiency demands of 6G networks. For instance, PoW and PoS often rely on chain-like structures for transaction recording, limiting their processing capabilities in high-concurrency environments. Moreover, as the number of nodes increases in large-scale networks, energy consumption and latency issues become more pronounced. Therefore, in order to better apply blockchain technology in the 6G network environment, the development of new consensus protocols is necessary to cater to the requirements of high-speed network environments.

**Table 1:** Comparative analysis of traditional consensus protocols and DAG consensus protocols.

Protocol	Throughput	Latency	Scalability	EE	Communication	Consistency	Structure
POW	Low	High	Low	Low	Synchronous	Strong	Chain
POS	Low	High	Low	Low	Synchronous	Strong	Chain
PBFT	Moderate	Moderate	Low	Moderate	Semi-Synchronous	Strong	Chain
DAG	High	Low	High	High	Asynchronous	Final	Graph

In contrast, DAG-based consensus protocol offers numerous advantages, including high concurrency, low latency, and high throughput, which align well with the requirements of 6G networks. The asynchronous communication model and non-linear transaction confirmation method employed by DAG consensus significantly enhance system throughput and reduce latency, while also providing better scalability and energy efficiency(EE) [33]. DAG consensus allows for parallel processing of multiple transactions, overcoming the bottlenecks associated with traditional chain-like structures. Furthermore, DAG adopts a finality consistency model, ensuring data correctness while processing transactions more rapidly. However, this optimization may entail sacrificing some immediate determinism in extreme cases. Nevertheless, in many real-time scenarios where high immediacy is required and a certain degree of time lag in consistency is acceptable, DAG consensus protocols demonstrate their unique advantages. Therefore, the DAG-based consensus protocol holds promise as a favorable choice for 6G networks, particularly in supporting high-concurrency applications.

**Fig 2:**
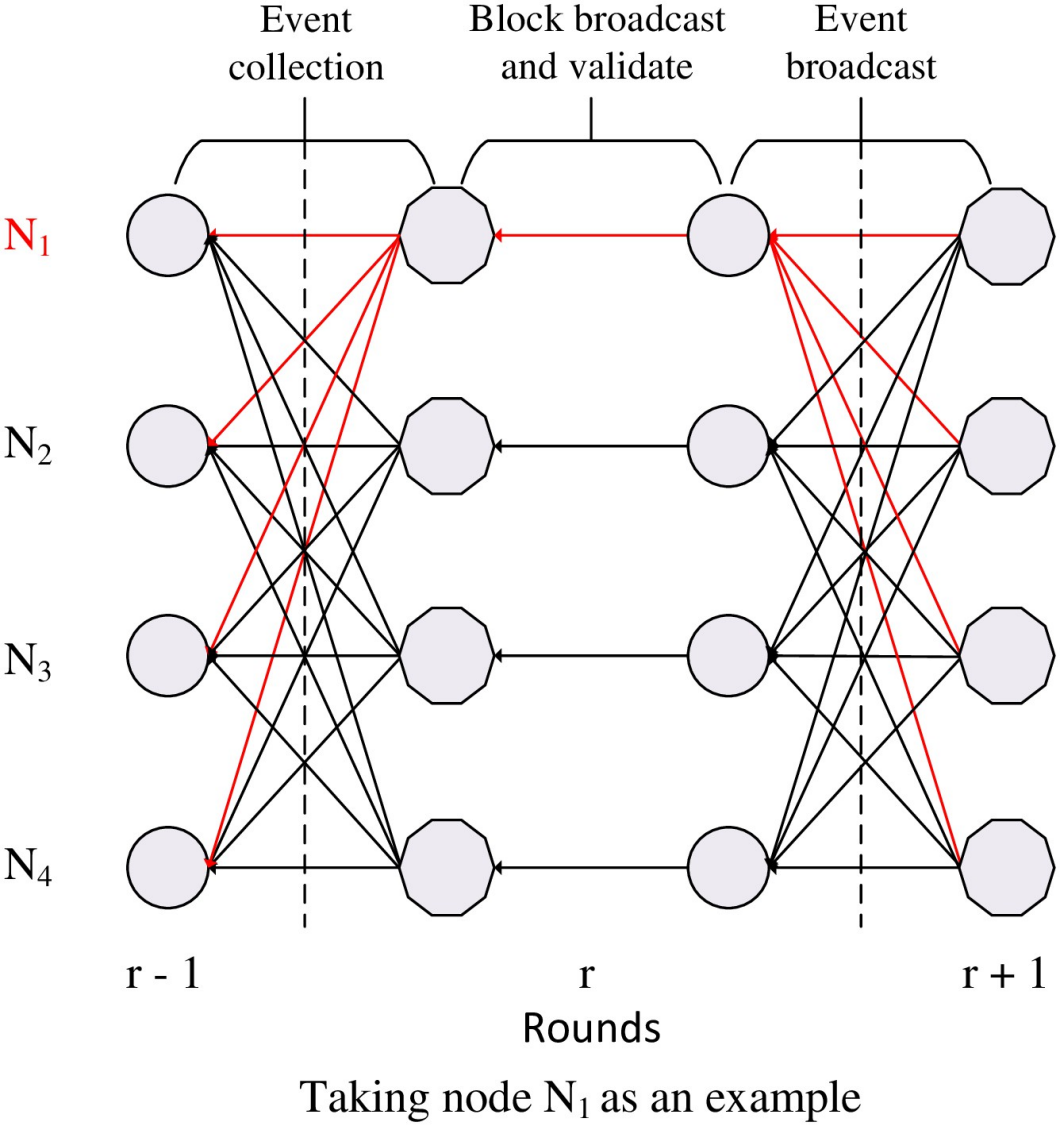
The round-based DAG structure.

Based on the aforementioned considerations and to better adapt to real-world asynchronous network environments with network communication failures and unpredictable network conditions, we propose a solution for a DAG-based asynchronous consensus protocol in blockchain. Additionally, we employ sharding techniques to further enhance transaction throughput and reduce the burden on participating nodes. The blockchain network is divided into multiple shards that concurrently process different sets of transactions. Within each shard, nodes maintain a consistent DAG structure based on rounds (constants) locally. In each round, two types of messages are broadcasted: blocks containing specific transactions and events containing block hashes. These events serve to prove the legitimacy of blocks and facilitate inter-shard communication. When a node receives a sufficient number of events for the current round, it proceeds to the next round and creates a new block, which is then broadcasted to neighboring nodes within the same shard. Nodes receiving the block validate it, and once the block is validated by a majority of nodes, the node that created the block generates a corresponding event to prove its legitimacy. The DAG structure is illustrated in Fig 2.

**Fig 3:**
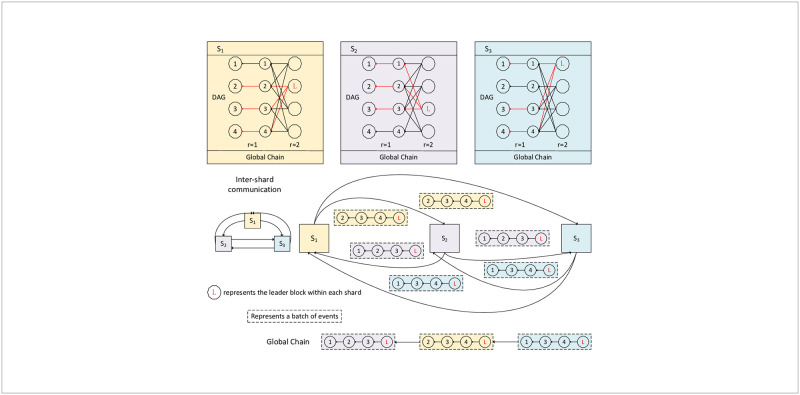
The architecture of the consensus protocol.

Specifically, within each shard, nodes employ a structured DAG to facilitate parallel broadcasting and transaction processing. Nodes only broadcast and store transaction blocks of their local shard, thereby enhancing transaction processing efficiency and reducing storage costs. To ensure intra-shard consistency, we introduce a randomized approach to order the blocks within the shard. In more detail, we utilize an active threshold signature scheme [34] to obtain shard-consistent random coins, which are used to determine leader blocks. By sorting the ancestor blocks of the leader block, we establish a consistent block order within the shard. Furthermore, to ensure global consistency, inter-shards consensus is required, nodes send the batch of events corresponding to the blocks that have been confirmed in the intra-shard consensus to the nodes within other shards through inter-shards communication, instead of the blocks themselves. This significantly reduces inter-shard communication overhead. Additionally, batch merging reduces network bandwidth consumption and accelerates confirmation speed. Through this approach, nodes in different shards can maintain a consistent global block event state, effectively addressing the security downgrade caused by sharding. The architecture of the consensus mechanism is illustrated in Fig 3.

**Fig 4:**
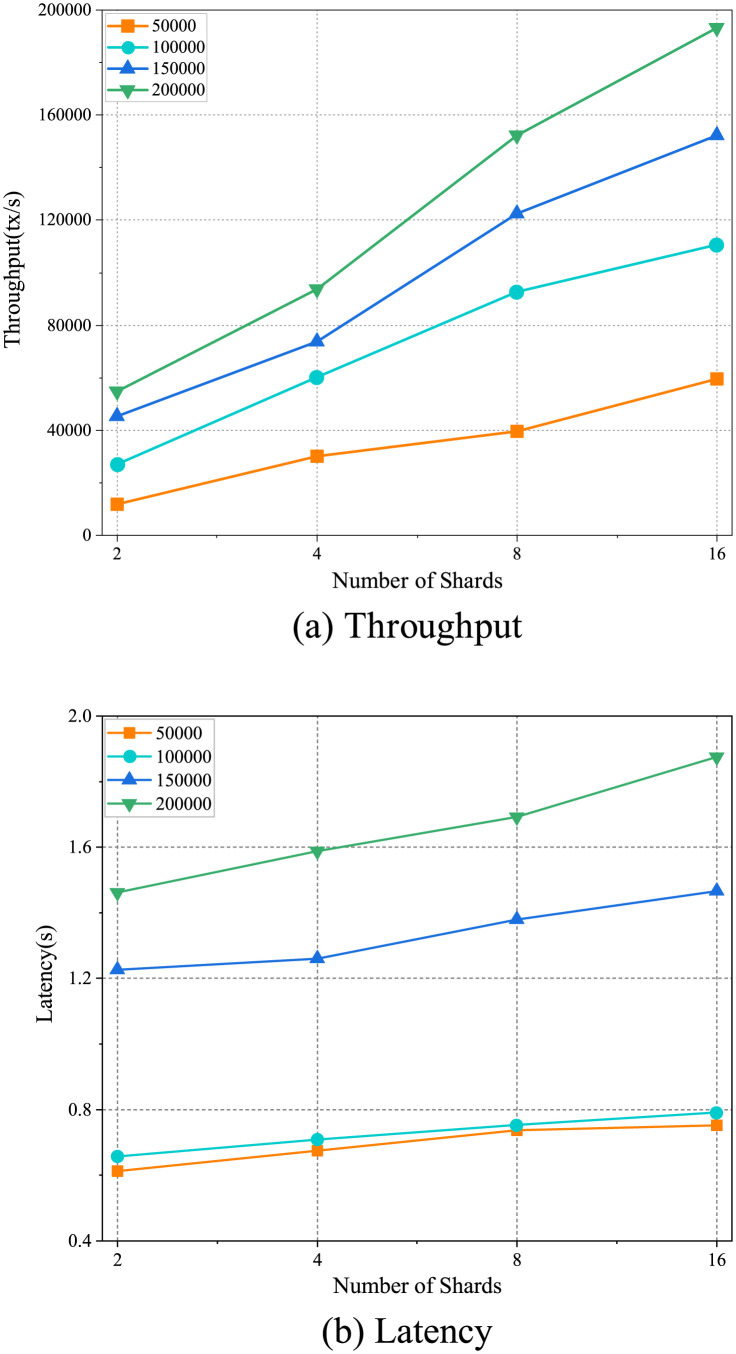
Performance of the protocol with different number of shards and different input rates. (Data presented in S1 and S2 Tables in S1 File).

We have set up a local experimental environment for evaluating the performance of the consensus protocol. This experimental environment consists of four high-performance servers, each equipped with an Intel(R) Xeon(R) 5218R CPU running at 2.10GHz, 128GB of memory, and 40 CPU cores, running the Linux Ubuntu Server 20.04 operating system. To evaluate its performance in scenarios involving a large number of transactions and high workloads, we conducted tests on the protocol’s performance under different numbers of shards and input rates. The experimental results, shown in Fig 4, depict the protocol’s throughput and latency for configurations with 2, 4, 8, and 16 shards, as well as input rates of 50,000 tx/s, 100,000 tx/s, 150,000 tx/s, and 200,000 tx/s. Here, the input rate represents the rate at which clients submit transactions to the system. The experimental results demonstrate a significant improvement in throughput and effective control of latency as the number of shards and input rate increase. Particularly, in the configuration with 16 shards and an input rate of 200,000 tx/s, the protocol achieves a throughput of over 190,000 tx/s with a latency of less than 2 seconds. The findings indicate that the protocol exhibits excellent adaptability and performance in future 6G environments characterized by large-scale and high workload scenarios.

### Optimization strategies for blockchain storage and communication based on 6G networks

Given the high data rates and large-scale data processing requirements of 6G networks, effective optimization of storage and communication of blockchain systems is essential. Taking into account the characteristics of 6G networks, we have devised a set of strategies to optimize storage and communication. These strategies are aimed at ensuring that the blockchain system performs well and remains stable in high-load and large-scale transaction scenarios.

#### Storage optimization

To support the vast storage demands of blockchain, data compression technology emerges as a crucial means. In scenarios where blockchain storage entails a massive amount of data, data compression techniques can significantly reduce the occupancy of storage space. Moreover, with the advancement of bandwidth and transmission speeds in 6G networks, compression algorithms can better facilitate the application of blockchain technology. For instance, by utilizing compression algorithms, large-scale blockchain data files can be compressed into smaller ones, thereby conserving storage space. Furthermore, compression techniques can also be employed to compress transaction data, reducing the volume of stored and transmitted data, thereby further enhancing system efficiency and performance.

Furthermore, in blockchain technology, sharding is a common method of data storage optimization. By dividing the blockchain data into multiple shards, the amount of data that each node needs to store can be reduced. To ensure load balancing in storage, the size and quantity of shards can be dynamically adjusted based on the node’s workload. When a node needs to access data from other shards, it can request the data from other nodes and perform merging operations. However, to maintain data consistency, certain synchronization operations may be required.

#### Communication optimization

To optimize P2P communication, attention should be given to both node selection and message routing. Regarding node selection, a comprehensive evaluation of node quality can be conducted, taking into account factors such as network topology, bandwidth, and response time. Prioritizing high-quality nodes in this process can effectively reduce message transmission latency and enhance the overall system efficiency. For instance, algorithms like Distributed Hash Tables (DHT) can be utilized to identify the most optimal nodes. In terms of message routing, more efficient routing can be achieved by employing distance-based routing algorithms such as Kademlia [35] and other related technologies, enabling rapid identification of the nearest nodes and improving message transmission efficiency.

## Future research directions

Here, we propose several research directions aimed at promoting the deep integration of blockchain and 6G networks, and further enhancing network efficiency, security, and scalability.

### The combination of intelligent blockchain and 6G network

The future 6G network will no longer be merely an infrastructure for device connectivity but a more intelligent and adaptive network. This implies that the integration of blockchain and 6G networks will require even more intelligent approaches. Therefore, future research directions should explore the combination of blockchain technology and AI to achieve a more intelligent and adaptive integration of blockchain and 6G networks. One research direction is to leverage machine learning techniques to enhance the selection and data processing of blockchain nodes, enabling more efficient communication and data storage. Furthermore, the application of blockchain technology and AI can be explored to achieve more intelligent network management and security mechanisms, thereby enhancing the security and reliability of the network.

### Flexible and efficient blockchain consensus protocol

In the realm of 6G networks, the consensus protocol of blockchain necessitates meeting heightened performance requirements to facilitate expedited transaction processing and large-scale data transmission. Concurrently, given the multidimensional architecture of 6G networks encompassing space, aerial, terrestrial, underground, and underwater domains, security and privacy concerns become more intricate and pivotal. Hence, future research endeavors can converge on devising consensus protocols that are more secure, efficient, and privacy-preserving, aligning with the demands of 6G networks. Pertinent research areas encompass, but are not limited to, leveraging cryptographic techniques to enhance the resilience and interference resistance of consensus protocols, crafting innovative consensus protocols tailored to the 6G network architecture, and exploring avenues to expedite blockchain transactions and data processing while upholding stringent security prerequisites.

### Post-quantum era security safeguards

With the advancements in quantum computing technology, traditional public key encryption algorithms have become vulnerable to attacks, posing significant security challenges to the practical application of blockchain technology and 6G networks. Therefore, exploring the integration of blockchain and 6G networks with quantum security technology to enhance network security is an important research direction. Specifically, the use of encryption methods based on quantum key distribution can be explored to protect the communication and storage processes of blockchain, thereby improving its security. Additionally, researchers can investigate leveraging quantum technology to enhance the randomness and security of blockchain, such as using quantum random number generators to generate high-quality random numbers, thereby enhancing the strength and security of cryptography.

Furthermore, addressing the challenges posed by the continually evolving complex network environments and dynamic load conditions, deepening the research on the adaptability and stability of consensus algorithms is crucial. This encompasses, but is not limited to, developing more intelligent dynamic sharding strategies to balance system loads, designing flexible and efficient adaptive parameter adjustment mechanisms, and exploring the integration of Trusted Execution Environments (TEEs) and other emerging hardware technologies into consensus algorithms to enhance the security and efficiency of the consensus process. Additionally, within the broader context of the blockchain ecosystem increasingly emphasizing interoperability, innovation in consensus algorithms for cross-chain communication scenarios emerges as one of the core trends for future development. By studying consensus mechanisms applicable to interactions between different blockchains, it becomes possible to effectively promote value exchange, information dissemination, and resource coordination, thereby driving the interconnectedness of the entire blockchain ecosystem. Furthermore, to expand the scope of blockchain technology applications and the range of service recipients, in-depth exploration of the design and implementation methods of lightweight clients is particularly crucial. This lightweight participation model enables devices with limited resources to easily access blockchain networks, not only lowering the user threshold but also providing strong support for the widespread adoption and deep integration of blockchain technology in various fields such as healthcare, IoT, and smart cities, thus truly achieving its broad dissemination and deep integration in the 6G network environment.

## Conclusion

The present manuscript presents a method based on the design of security mechanisms and performance optimization to achieve a secure and efficient integration of blockchain and 6G networks. Initially, we delve into the application scenarios of 6G and the empowerment of blockchain in the 6G context, conducting a detailed analysis of the advantages of 6G networks and the synergistic effects of blockchain and 6G. Subsequently, we have explored a security mechanism suitable for the integration of blockchain and 6G networks. Through a detailed analysis of security threats and attack types, we have conducted an in-depth study of the design requirements for security mechanisms in this combined scenario. Furthermore, we introduce a DAG-based blockchain asynchronous consensus solution, aiming to fulfill the desired characteristics of 6G networks. Additionally, we provide optimization strategies pertaining to storage, communication to better cater to the extensive data exchange and processing requirements in 6G networks. Lastly, we offer valuable research directions to guide the future development of the integration between blockchain and 6G networks.

## Supporting information

S1 File Towards secure and efficient integration of blockchain and 6G networks. (DOCX)
