# Supplementary material for: Towards secure and efficient integration of blockchain and 6G networks
Source: PLoS One. 2024 Apr 11;19(4):e0302052. doi: 10.1371/journal.pone.0302052 (PMC11008884; doi:10.1371/journal.pone.0302052)
Supplement: S1 File — (DOCX) [file pone.0302052.s001.docx]

**Towards secure and efficient integration of blockchain and 6G networks**

**Supplementary Materials**

**Table S1** Throughput(tx/s) of the protocol with different number of shards and different input

rates, presented in Fig 4(a).

| Number of shards | Input rate(tx/s) | | | |
| --- | --- | --- | --- | --- |
|  | 50000 | 100000 | 150000 | 200000 |
| 2 | 11824 | 26908 | 45432 | 54764 |
| 4 | 30047 | 60108 | 73624 | 93700 |
| 8 | 39616 | 92608 | 122448 | 152288 |
| 16 | 59488 | 110816 | 152144 | 193272 |

**Table S2** Latency(s) of the protocol with different number of shards and different input

rates, presented in Fig 4(b).

| Number of shards | Input rate(tx/s) | | | |
| --- | --- | --- | --- | --- |
|  | 50000 | 100000 | 150000 | 200000 |
| 2 | 0.613 | 0.658 | 1.227 | 1.462 |
| 4 | 0.675 | 0.709 | 1.26 | 1.588 |
| 8 | 0.738 | 0.753 | 1.379 | 1.693 |
| 16 | 0.752 | 0.792 | 1.466 | 1.875 |
